# Mealybug (Hemiptera: Pseudococcidae) Species Associated with Cacao Mild Mosaic Virus and Evidence of Virus Acquisition

**DOI:** 10.3390/insects12110994

**Published:** 2021-11-04

**Authors:** Alina S. Puig, Sarah Wurzel, Stephanie Suarez, Jean-Philippe Marelli, Jerome Niogret

**Affiliations:** 1 Subtropical Horticultural Research Station, USDA-ARS, Miami, FL 33158, USA; sarah.b.wurzel@gmail.xn; 2 Mars Wrigley, Miami, FL 33158, USA; stephaniensuarez@gmail.com; 3 Mars Wrigley, Davis, CA 95616, USA; jean-philippe.marelli@effem.com; 4 Mars Wrigley, James Cook University, Smithfield, QLD 4878, Australia; jerome.niogret@effem.com

**Keywords:** DNA barcoding, molecular markers, *Pseudococcus*, *Maconellicoccus hirsutus*, mealybug, cacao, *Badnavirus*, virus vector, Florida

## Abstract

**Simple Summary:**

Cacao mild mosaic virus was discovered in Trinidad in 1943, where it was shown to be transmitted by five mealybug species. It was recently detected in Puerto Rico, Brazil, and the USA; however, no information is available on insect vectors in these locations. Mealybugs belong to a diverse group known as Pseudococcidae, and species’ composition differs among geographic regions. A study conducted on infected trees in Florida found four species of mealybug present: *Pseudococcus jackbeardsleyi*, *Maconellicoccus hirsutus*, *Pseudococcus comstocki*, and *Ferrisia virgata*. Of these, *P. jackbeardsleyi* and *M. hirsutus* have not been tested for their ability to transmit viruses to cacao. Cacao mild mosaic virus was detected in 34.6 to 43.1% of the insects tested; however, acquisition did not differ among species. Owing to their prevalence (>72%), transmission studies should be conducted to determine the ability of *P. jackbeardsleyi* and *M. hirsutus* to transmit the virus. This research improves our understanding of the mealybugs associated with virus-infected plants in Florida and identifies potential new insect vectors. Knowledge of vector species is essential for selecting the most effective control strategies and minimizing disease spread.

**Abstract:**

*Theobroma cacao* is affected by viruses on every continent where the crop is cultivated, with the most well-known ones belonging to the *Badnavirus* genus. One of these, cacao mild mosaic virus (CaMMV), is present in the Americas, and is transmitted by several species of Pseudococcidae (mealybugs). To determine which species are associated with virus-affected cacao plants in North America, and to assess their potential as vectors, mealybugs (*n* = 166) were collected from infected trees in Florida, and identified using COI, ITS2, and 28S markers. The species present were *Pseudococcus jackbeardsleyi* (38%; *n* = 63), *Maconellicoccus hirsutus* (34.3%; *n* = 57), *Pseudococcus comstocki* (15.7%; *n* = 26), and *Ferrisia virgata* (12%; *n* = 20). Virus acquisition was assessed by testing mealybug DNA (0.8 ng) using a nested PCR that amplified a 500 bp fragment of the movement protein–coat protein region of CaMMV. Virus sequences were obtained from 34.6 to 43.1% of the insects tested; however, acquisition did not differ among species, X^2^ (3, *N* = 166) = 0.56, *p* < 0.91. This study identified two new mealybug species, *P. jackbeardsleyi* and *M. hirsutus*, as potential vectors of CaMMV. This information is essential for understanding the infection cycle of CaMMV and developing effective management strategies.

## 1. Introduction

Mealybugs (Hemiptera: Pseudococcidae) are phloem feeders that use long, slender mouthparts to uptake plant fluids [1], which reduces the vigor of host plants. They can feed on all plant tissues, and severe infestations cause defoliation and, eventually, plant death. Some species inject plant toxins during feeding, causing twisted/stunted growth [2]. The damage generated varies among taxa and is determined by their reproductive potential, temperature tolerance, preferred feeding locations, the existence of effective control strategies, and their ability to transmit viruses [3].

On cacao, mealybugs are economically important owing to their role as virus vectors [4]. Viruses are present on every continent where cacao is grown commercially, most of which belong to the Badnavirus genus. At least 11 Badnavirus species are known to infect cacao, some of which cause reduced yield or tree death [5]. Most research focuses on highly virulent viruses in West Africa associated with cacao swollen shoot disease (CSSVD). Infected trees experience severe yield decline and death within a few years [6,7]. Annual production loss due to CSSVD was estimated to be 76,000 metric tons in 2012 [8].

In the Americas, virus symptoms were reported on cacao in 1944 in Trinidad and Venezuela [9,10]. Research in Trinidad described two distinct strains, which were recently characterized and named cacao mild mosaic virus (CaMMV) and cacao yellow vein banding virus (CYVBV) [11]. These viruses were thought to be relatively rare, until CaMMV was detected in Puerto Rico [12] and Brazil [13], and then intercepted in material under quarantine in the USA [14] in the past couple of years. Trees infected with CaMMV experience branch dieback and annual yield reduction of 6.6–19%, comparable to mild to moderate strains of CSSV in West Africa [15,16]. Tree death due to infection with CaMMV has not been confirmed.

Isolates of CaMMV that have been molecularly characterized to date have genome sizes just over 7500 bp and are made up of four open reading frames (ORFs); ORFs1–3 and ORFY [11,13]. Although virus particles have not yet been detected in infected tissue, they are assumed to be bacilliform, made up of a double-stranded DNA genome, as is characteristic of members of the Badnavirus genus [17]. Badnaviruses are transmitted to cacao by multiple mealybug species [6,11]; however, owing to the high diversity of Pseudococcidae, each geographic region has a different species composition. In West Africa, *Pseudococcus njalensis* (Laing) is the main vector, owing to its abundance in the area [18], while *Planococcus citri* (*Pl. citri*) (Risso) is the most significant vector of cacao virus in Trinidad, where it was shown to be capable of transmitting both virus species present, CaMMV and CYVBV [4].

Numerous factors influence the importance of each species as virus vectors; that is, prevalence, mobility, uptake efficiency, and feeding preferences. For example, lower transmission rates of cacao swollen shoot virus (CSSV) with *Ferrisia virgata* (Cockerell) than with *Pseudococcus njalensis* were attributed to differences in feeding behavior, where *F. virgata* fed directly from phloem less frequently [19]. Detailed feeding studies showed that *F. virgata* frequently ingested sap from xylem tissue, where virus titer is presumed to be lower.

Successful transmission requires mealybugs to uptake the virus from the phloem during feeding and, subsequently, deposit it in the phloem of a different plant [19]. The ability to transmit viruses varies among related species. For example, *Pseudococcus longispinus* can transmit two strains of CSSV to cacao, but cannot transmit either CaMMV or CYVBV, the two cacao-infecting viruses present in Trinidad [20]. Mealybugs can acquire CaMMV, and are infectious, after as little as 33 min of feeding on infected material. Infectivity can persist in up to 23 h in *P. citri* and eight hours in *D. brevipes* following separation from the infected plant [20].

Previous studies showed that cacao infecting Badnaviruses generally have semi-persistent transmission characteristics [20]. However, circulative transmission has been reported in *P. njalensis*, with CSSV being transmitted after a molt [21]. Overall, these viruses have both non-persistent (stylet-borne) and persistent (circulative) characteristics [22]. 

**Figure 1:**
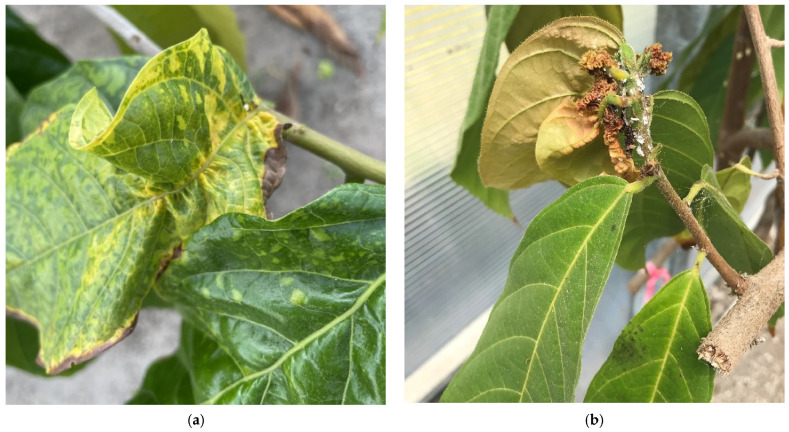
Signs of virus infection and mealybug infestation on *Theobroma cacao*: (**a**) leaves with mosaic symptoms characteristic of cacao mild mosaic virus and mild distortion caused by *Maconellicoccus hirsutus*, and (**b**) severe bunchy top caused by *M. hirsutus*.

In Trinidad, where CaMMV was first discovered, the virus was shown to be transmitted by five mealybug species: *Pl. citri, Dysmicoccus brevipes, D. sp. near brevipes, Ferrisia virgata*, and *Pseudococcus comstocki*. However, no research has been done on this in nearly 70 years. Vector information is not available from other countries, as CaMMV was only recently detected outside of Trinidad [12]. The purpose of this study was to identify mealybug species associated with cacao plants infected with CaMMV in Florida, USA (Figure 1), and to determine their ability to acquire the virus. This was done by identifying specimens using genetic markers and assaying individuals for the presence of CaMMV using a nested PCR. This information is essential for understanding the infection cycle of CaMMV and identifying potential new vectors.

## 2. Materials and Methods

### 2.1. Insect Sampling and DNA Extraction 

In January 2021, female mealybugs were collected from 23 randomly selected *Theobroma cacao* trees in Florida confirmed to be infected with CaMMV following nested PCR testing, as described in Puig [14], followed by Sanger sequencing of the amplicons. Specimens were collected from the pods, stems, leaves, and flowers of each tree (up to 5 individuals from each tissue × tree combination), and placed in microcentrifuge tubes containing 70% ethanol. DNA was extracted from individual specimens using the Qiagen DNeasy Blood and Tissue Kit using a shortened, 10 min lysis step, as described in Albo et al. [23]. The final resuspension step was done with 50 µL of elution buffer for adults and 30 µL for the smaller nymphs. DNA was quantified using a Qubit 4 Fluorometer and the 1× dsDNA High Sensitivity Assay Kit (Life Technologies Corp., Carlsbad, CA, USA).

### 2.2. Mealybug Identification

**Figure 2:**
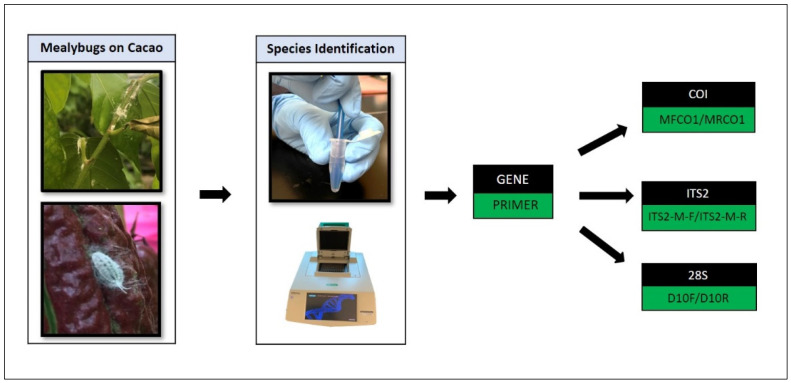
Mealybugs feeding on virus-infected trees of *Theobroma cacao* were identified using COI, ITS2, and 28S markers amplified and sequenced using MFCO1/MRCO1, ITS2-M-F/ITS2-M-R, and D10F/D10R primers.

To identify mealybugs, the cytochrome c oxidase subunit I (COI) region, the internal transcribed spacer region (ITS2), and the ribosomal DNA subunit 28S (28S) were amplified and sequenced using the primer pairs, MFCO1/MRCO1, ITS2-M-F/ITS2-M-R, and D10F/D10R, respectively (Figure 2; Table 1). PCRs were performed in 25 μL volumes, consisting of 12.5 µL 2× Immomix Red (Bioline Reagents Ltd., London, UK), 1 µL each of 10 µM forward and reverse primers, 1 µL of DNA template, and 9.5 µL sterile nuclease-free water, as presented in Puig et al. [24].

Amplification was visualized on a 1% (*w*/*v*) agarose gel at 150 V for 35 min. PCR products were purified with Qiagen PCR Purification Kit (Hilden, Germany) and sent to Eurofins for Sanger sequencing. Sequences were edited and aligned using Geneious^®^11.1.2 (Biomatters Ltd., Auckland, New Zealand), and analyzed in BLASTn for identification. Specimen identification was determined based on BLASTn results of the COI sequences because this genetic region is considered the most biologically informative. The samples from which COI sequences were not obtained were identified by aligning their ITS2 or 28S sequences with corresponding sequences from samples that had been identified using COI. A representative subset of sequences generated in this study was deposited in GenBank and is publicly available (Supplementary File S1). A chi-square test of independence was used to determine whether there was a significant association between mealybug species and the tissue on which they were found (flower, leaf, pod, and stem), using the PROC FREQ in SAS Ver. 9.3 (SAS Institute, Cary, NC, USA).

### 2.3. Detection of CaMMV in Mealybugs

Presence of CaMMV was determined by running a nested PCR for each sample with virus-specific primers (Table 2) [14]. The initial reaction was 20 μL with 10 μL Sigma-Aldrich© JumpStart™ REDTaq^®^ ReadyMix™ (Sigma-Aldrich, St Louis, MO, USA), 7.6 μL molecular grade water, 0.8 μL of each 10 μM primer (Mia1145F and Mia1926R), and 0.8 ng of DNA template. Amplification conditions were an initial 2 min step at 94 °C followed by 22 cycles of 94 °C (25 s), 34 °C (25 s), and 72 °C (1 min), and then a final extension at 72 °C for 12 min.

The final reaction was also a 20 μL volume, as described above, with primers Mia1396F and Mia1876R and 0.8 μL of amplified PCR product as the template. Amplification conditions were an initial 2 min step at 94 °C followed by 37 cycles of 94 °C (20 s), 53 °C (20 s), and 72 °C (1 min), and then a final extension at 72 °C for 10 min. Amplified PCR products were visualized, purified, and sequenced as described above. Sequences were aligned and edited using Geneious^®^11.1.2 and analyzed in BLASTn for identification. The number of specimens of each species from which CaMMV was amplified and sequences was analyzed using a chi-square test of independence, as described previously, to determine whether there was a significant association between mealybug species and virus acquisition.

### 2.4. Phylogenetic Comparison

To determine the diversity of virus strains detected in mealybugs, a phylogenetic analysis was performed with MP–CP sequences in MEGA X [28], following alignment using the MUSCLE algorithm [29]. The Kimura two-parameter model was chosen using the MEGA X models function to assess various nucleotide substitution models [30]. Initial trees for the heuristic search were generated by applying neighbor-join and BioNJ algorithms to a matrix of pairwise distances estimated using the maximum composite likelihood (MCL) approach, and then choosing the topology with a superior log likelihood value. The rate variation model allowed for some sites to be evolutionarily invariable ([+*I*], 42.48% sites). Tree branch lengths were measured in the number of substitutions per site.

Codon positions included were first + second + third + noncoding, and positions with less than 95% site coverage were eliminated. Fewer than 5% alignment gaps, missing data, and ambiguous bases were allowed at any position (partial deletion option). There were 60 nucleotide sequences and 401 positions in the final dataset.

## 3. Results

### 3.1. Insect Sampling and DNA Extraction

A total of 170 mealybugs were collected from pods, leaves, stems, and flowers of cacao trees in a Florida greenhouse. These trees were determined to be infected with CaMMV following nested PCR testing of three leaves per plant, followed by Sanger sequencing of amplified fragments. The detection of multiple virus strains in several trees suggests that mixed infections are common [31]. Four of these were discarded owing to low DNA concentrations and omitted from subsequent analyses. Three of the 20 selected trees that had no mealybugs present were omitted and were replaced with three additional, randomly selected trees. No mealybugs were found on the tree roots inspected. 

### 3.2. Mealybug Identification

Sequences of all three markers yielded consistent species matches for *Pseudococcus comstocki* (Kuwana) (*n* = 26) and *Maconellicoccus hirsutus* (*n* = 55), with coverage and identities ranging from 96 to 100% (Table 3). For *Pseudococcus jackbeardsleyi* (Beardsley) (*n* = 65) and *Ferrisia virgata* (*n* = 20), only COI sequences provided unambiguous identification. 28S sequences of both species were close matches to multiple genera available in GenBank. In contrast, no close matches were found for the ITS2 sequences obtained from *P. jackbeardsleyi*.

*Pseudococcus jackbeardsleyi* was the predominant species found in this study, comprising 38% (*n* = 63) of all insects collected. It was followed by *M. hirsutus*, which made up over 34% of the specimens examined. Most mealybugs were collected from stems (*n* = 57), followed by pods (*n* = 46) (Table 4). A chi-square test of independence showed that there was a significant association between mealybug species and the tissue on which they were found. The proportion of specimens found on flowers, leaves, pods, and stems differed by species, X^2^ (9, *N* = 166) = 20.2, *p* < 0.02.

### 3.3. Detection of CaMMV in Mealybugs

**Figure 3:**
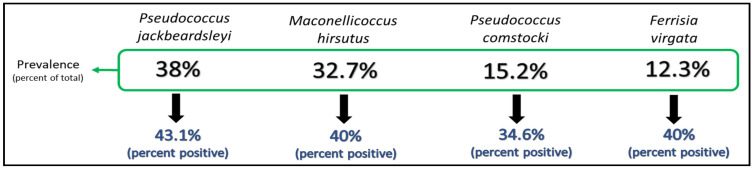
Mealybug species collected from virus-infected cacao trees, listed (left to right) in decreasing order of relative abundance (prevalence). *Cacao mild mosaic virus* sequences were obtained from 34.6 to 43.1% of insects collected.

To determine the relative rates of virus acquisition, nested PCR was carried with DNA of 166 mealybugs collected from 20 cacao trees infected with CaMMV. Acquisition was determined based on the amplification of a ~500 bp fragment and homology of the resulting sequence to previously obtained CaMMV sequences. Sixty-seven of all mealybugs (40.4%) tested positive for CaMMV, with acquisition rates ranging from 34.6% in *F. virgata* to 43.2% in *P. jackbeardsleyi* (Figure 3). A chi-square test of independence showed that there was no significant association between mealybug species and virus acquisition. The proportion of specimens with detectable levels of CaMMV did not differ by species, X^2^ (3, *N* = 166) = 0.56, *p* < 0.91.

### 3.4. Phylogenetic Comparison

**Figure 4:**
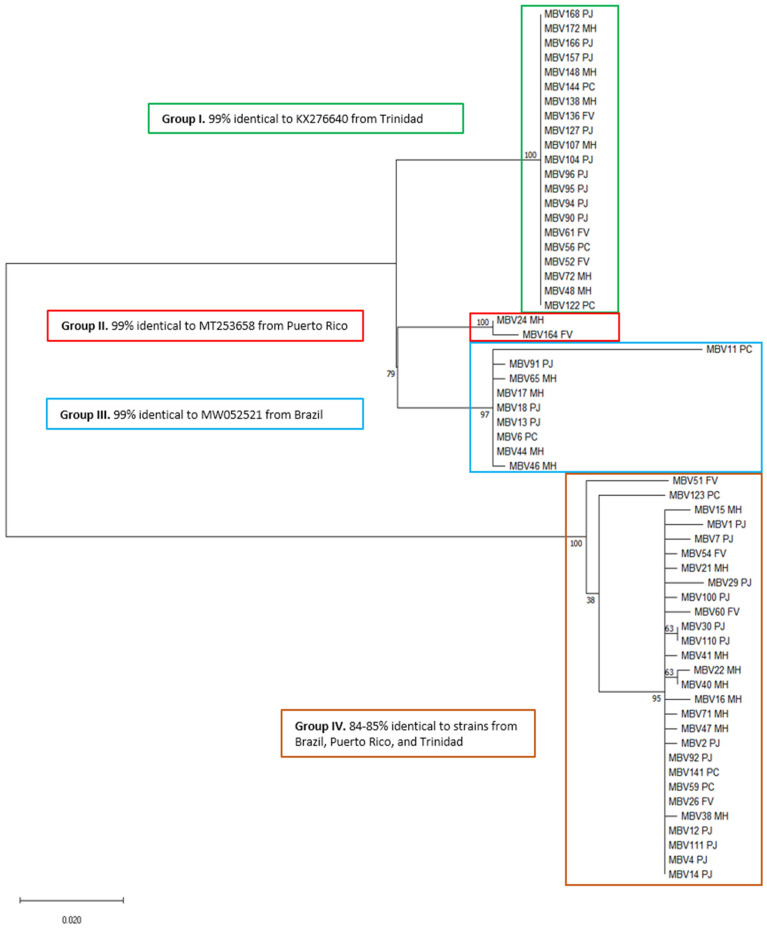
Phylogenetic tree constructed using the nucleotide sequences of the coat protein region of *cacao mild mosaic virus*. Sequence names are followed by the initials of the mealybug species in which they were found; *P. jackbeardsleyi* (PJ), *P. comstocki* (PC), *M. hirsutus* (MH), and *F. virgata* (FV).

The CaMMV sequences (*n* = 60) generated in this study clustered into four primary groups. Groups I, II, and III shared approximately 99% sequence identity to isolates from Trinidad, Puerto Rico, and Brazil, respectively, as determined following BLASTn analysis (Figure 4). Group IV is the largest (*n* = 28), and shared approximately 84 to 85% nucleotide sequence identity with strains from Brazil, Puerto Rico, and Trinidad. Group I was the most genetically uniform, with no nucleotide differences among the 21 sequences. A representative subset of sequences generated in this study was deposited in GenBank and is publicly available (Supplementary File S1). 

## 4. Discussion

This study identified two new mealybug species, *P. jackbeardsleyi* and *M. hirsutus*, as potential vectors of CaMMV. *P. jackbeardsleyi* has been frequently collected throughout Florida (USA), the Caribbean, and Central and South America on various crops, and is thought to be native to the area. It has only been reported on cacao in Colombia [32]. However, it was described as a species relatively recently, in 1996, and is commonly misidentified as *P. elisae* or *P. landoi* [33]. In contrast, *M. hirsutus* is native to southeast Asia, but has been highly invasive following its introduction into the Caribbean in 1994. Successful control has been obtained in some locations using parasitoid and predator species only when they were released quickly after initial detection [34]. It was introduced to the state of Florida nearly 20 years ago and has become widespread, affecting fruit trees such as papaya, citrus, and soursop [35,36]. *M. hirsutus* has been reported to affect cacao in Trinidad [37] and Brazil [38].

These species have been reported to affect cacao in Africa, the Caribbean, and South America [37,38,39], but this is the first report of them affecting cacao in Florida. Neither species has been tested for their ability to transmit viruses to cacao, but they are closely related to confirmed vectors, and have been detected on CSSV-infected cacao in Cote d’Ivoire [39]. Most research on mealybugs affecting *T. cacao* is done in West Africa, where the more damaging, and economically significant, viruses are found. However, owing to the status of CaMMV as an emerging disease in the Western hemisphere, it is essential to determine the transmission routes and other significant epidemiological variables.

Cytochrome c oxidase subunit I (COI) is considered the most informative marker for insects; however, it has proven difficult to amplify in mealybugs [26,40]. The COI primers used in this study were designed by Wetten et al. [25] and validated on taxa collected from cacao in Asia, Africa, and the Americas (*Planococcus, Ferrisia, Dysmicoccus,* and *Pseudococcus*). They amplify a small section of the universal barcode region [41] that provided unambiguous identification in the species examined here.

The low homology between ITS2 sequences from *F. virgata* obtained in this study, and those available in GenBank, may indicate high genetic diversity, or the presence of cryptic species. In this study, ITS2 sequences were not considered informative for *P. jackbeardsleyi*, owing to the absence of these sequences in GenBank. However, the ITS2 sequences generated in this study were deposited in GenBank, making this marker valuable for future research.

The presence of multiple virus strains in mealybugs feeding on infected trees suggests that they are being actively transmitted in the greenhouse where this study took place. It is not clear how, or if, these strains interact with each other inside a given tree. Cross-protection has been reported with closely related strains, but does not always occur [42]. Transmission of CaMMV by mealybugs is followed by long latent periods (40–178 days), which reduces the effectiveness of rogueing to control disease [4].

Kirkpatrick [20] observed that *Pl. citri* formed large colonies on pods, but was less likely to feed on young stems or leaves. In contrast, *F. virgata* is the species most frequently observed to feed on leaves. The absence of *Pl. citri* in this study was surprising as this species is a common pest in Florida [43]. Accurate identification of species present in a population is essential for the selection of effective controls. Additional work on the preference of species for certain tissues and environmental conditions could provide useful information for management programs and insecticide applications.

## 5. Conclusions

The two most prevalent species, *P. jackbeardsleyi* and *M. hirsutus*, make up more than 72% of the mealybug population in this study. Prevalence in a population is a major characteristic affecting the significance of each species in the disease cycle. Neither species were mentioned in previous studies on cacao viruses in the Americas, suggesting a relatively recent association with *T. cacao*. Owing to their prevalence, transmission studies should be conducted to determine the ability of *P. jackbeardsleyi* and *M. hirsutus* to transmit CaMMV.

## Figures and Tables

**Table 1:** Genes, primer sequences, and amplicon sizes for the markers targeted in this study.

Gene	Primer	Sequence (5′–3′)	Amplicon Size (bp)	Reference
COI	MFCO1 MRCO1	ATATCTCAAATTATAAATCAAGAA ATTACACCTATAGATAAAACATAATG	379	[25]
ITS2	ITS2-M-F ITS2-M-R	CTCGTGACCAAAGAGTCCTG TGCTTAAGTTCAGCGGGTAG	~800	[26]
28S	D10F D10R	GTAGCCAAATGCCTCGTCA CACAATGATAGGAAGAGCC	738–767	[27]

**Table 2:** Primer pairs designed to amplify the movement protein–coat protein (MP–CP) genome region of CaMMV were used to detect the virus in individual mealybug specimens.

Gene	Primer	Sequence (5′–3′)	Amplicon Size (bp)	Reference
Cacao mild mosaic virus MP–CP	Mia1145F Mia1926R	YAACTTTGAGGACCAGATC YCTAAGTATCCARCTYCTTCCAAGR	806	[14]
Cacao mild mosaic virus MP–CP	Mia1396F	ACCGTGTCTAYCAGCACTGGA	503	[14]
	Mia1876R	CTGGRATWGCTCTTACKCCATGW		

**Table 3:** BLASTn results for COI, ITS2, and 28S sequences obtained in this study. Species determinations were made based on the best matches obtained with COI sequences. The results shown are from one representative of each species. Bold font denotes incorrect organisms among top matches (adapted from Puig et al. [24]).

	Marker	Genbank Match	GenBank No.	% Ident.	% Coverage
*Pseudococcus*	COI	*P. comstocki*	LC121496	98.9	100
*comstocki*	ITS2	*P. comstocki*	KU499509	96.3	100
	28S	*P. comstocki*	JF965413	99.8	98
*Pseudococcus*	COI	*P. jackbeardsleyi*	KJ187489	99.5	100
*jackbeardsleyi*	ITS2	***Pseudococcus viburni***	**KF819654**	**79.2**	**90**
	28S	***Pseudococcus viburni***	**AY427376**	**99.1**	**99**
		***Oracella acuta***	**JF965418**	**98.9**	**99**
		*P. jackbeardsleyi*	EU188510	99.9	95
*Maconellicoccus*	COI	*M. hirsutus*	MK090645	100	100
*hirsutus*	ITS2	*M. hirsutus*	KU883603	99.5	98
	28S	*M. hirsutus*	AY427403	99.5	96
*Ferrisia virgata*	COI	*Ferrisia virgata*	EU267205	99.2	100
	ITS2	*Ferrisia virgata*	KY423513	77.7	45
	28S	***P. comstocki***	**JF965413**	**97.6**	**99**
		***Ferrisia gilli***	**AY427398**	**99.1**	**98**
		*Ferrisia virgata*	AY427373	98.6	98

**Table 4:** Summary of mealybugs identified in this study and tissues from which they were collected.

Species	# on Pods	# on Leaves	# on Flowers	# on Stems	Total	%
*Maconellicoccus hirsutus*	25	14	5	13	57	34.3
*Pseudococcus jackbeardsleyi*	13	12	11	27	63	38.0
*Pseudococcus comstocki*	6	9	5	6	26	15.7
*Ferrisia virgata*	2	5	2	11	20	12.0
total	46	40	23	57	166	

## Data Availability

All data are available in the Supplementary File.

## Supplementary Materials

The following are available online at https://www.mdpi.com/article/10.3390/insects12110994/s1, Supplementary File S1: Nucleotide sequences submitted to GenBank.
